# Towards a more nuanced conceptualisation of differential examiner stringency in OSCEs

**DOI:** 10.1007/s10459-023-10289-w

**Published:** 2023-10-16

**Authors:** Matt Homer

**Affiliations:** School of Medicine, University of Leeds, Leeds, LS2 JT UK

**Keywords:** OSCE, Examiner stringency, Standard setting, Borderline regression

## Abstract

Quantitative measures of systematic differences in OSCE scoring across examiners (often termed examiner stringency) can threaten the validity of examination outcomes. Such effects are usually conceptualised and operationalised based solely on checklist/domain scores in a station, and global grades are not often used in this type of analysis. In this work, a large candidate-level exam dataset is analysed to develop a more sophisticated understanding of examiner stringency. Station scores are modelled based on global grades—with each candidate, station and examiner allowed to vary in their ability/stringency/difficulty in the modelling. In addition, examiners are also allowed to vary in how they discriminate across grades—to our knowledge, this is the first time this has been investigated. Results show that examiners contribute strongly to variance in scoring in two distinct ways—via the traditional conception of score stringency (34% of score variance), but also in how they discriminate in scoring across grades (7%). As one might expect, candidate and station account only for a small amount of score variance at the station-level once candidate grades are accounted for (3% and 2% respectively) with the remainder being residual (54%). Investigation of impacts on station-level candidate pass/fail decisions suggest that examiner differential stringency effects combine to give false positive (candidates passing in error) and false negative (failing in error) rates in stations of around 5% each but at the exam-level this reduces to 0.4% and 3.3% respectively. This work adds to our understanding of examiner behaviour by demonstrating that examiners can vary in qualitatively different ways in their judgments. For institutions, it emphasises the key message that it is important to sample widely from the examiner pool via sufficient stations to ensure OSCE-level decisions are sufficiently defensible. It also suggests that examiner training should include discussion of global grading, and the combined effect of scoring and grading on candidate outcomes.

## Introduction

There is a lot of quantitative evidence that examiners systematically vary in how they score in OSCEs (Bartman et al., 2013; Harasym et al., 2008; McManus et al., 2006; Yeates et al. 2021). This can be a serious threat to the validity of assessment outcomes, undermining the defensibility of the whole examination process. Most of this work focuses on a traditional conceptualisation of examiner stringency based solely on variation by examiner across domain or checklist totals scores in stations. It finds typically that there is considerable variation in scoring by examiners, which is therefore an ongoing threat to assessment reliability and wider validity of the assessment outcomes, and their use for high stakes purposes (Cook et al., 2015; Hatala et al. 2015; Ilgen et al., 2015).

Qualitative work investigating examiner decision-making (Wong et al., 2023; Yeates et al., 2013) has underlined the complexities of these processes, and of how individually situated these can be—for example highlighting different interpretations of scoring rubrics and idiosyncratic examiner behaviour. Ultimately, there is in the literature a serious questioning of the possibility of ‘objective’ measurement in OSCEs, and an argument for moving towards assessment systems in more naturalistic settings (Hodges, 2013). There is also some pushback against this suggestion with researchers advocating for the appropriate use of modern psychometric approaches in medical education (Pearce, 2020; Schauber et al., 2018).

Only occasionally has psychometrically-focussed examiner stringency research included variation in station-level global grades as part of the study (Homer, 2020, 2022). In the most recent work (Homer, 2022), a strong positive correlation (r = 0.76) between separate estimates of examiner stringency in scores and global grades was found, indicating a degree of consistency in examiner stringency measures across different scoring instruments—but far from perfect correlation, thereby suggesting a degree of differential stringency across instrument formats for individual examiners. The current paper builds on this work to investigate how examiner stringency might be better conceptualised and measured in two distinct ways: via the traditional approach to score stringency (based on differences across checklist/domain scores), but also in terms of how examiners might vary in this scoring across global grades (i.e. in their degree of discrimination in scores across global grades).

**Fig. 1:**
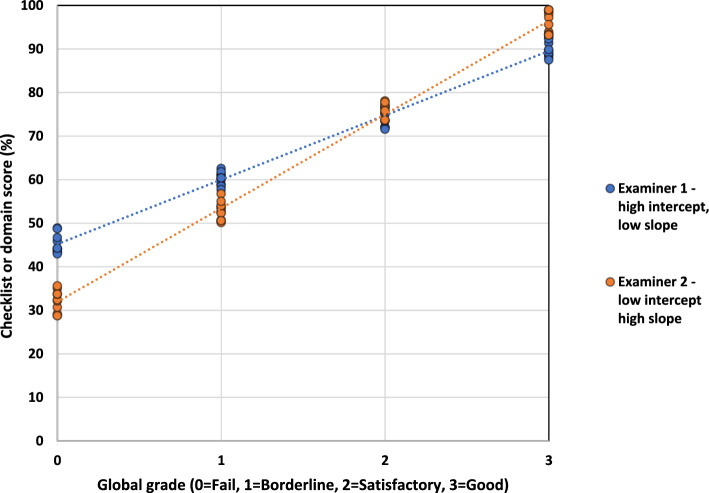
Schematic of two examiners with different stringencies

To illustrate visually these conceptualisations, Fig. 1 shows a hypothetical and idealized example of two sets of examiner scores/grades in a single station for the same set of candidates. Following the usual convention (Pell et al. 2010), global grades are plotted horizontally (i.e. on the x-axis), and total station percentage score is plotted vertically (y-axis). The line of best fit for each set of points is also drawn to make the difference in overall patterns of scoring especially clear.

Examiner 1 (blue) scores more highly at lower grades compared Examiner 2 (orange), and at higher grades the exact opposite is true. In simple linear regression parlance (Montgomery et al., 2012, Chapter 2), Examiner 1 has a relatively high intercept, but low slope (i.e. discrimination), and Examiner 2 has the opposite. By design, the two mean scores (and mean grades) by examiner in this hypothetical example are similar so traditional measures of examiner stringency based on scores or grades alone would not show much difference between them.

Differences in examiner behaviour obviously matter in a number of ways—crucially candidate outcomes are impacted (Homer, 2020, 2022; Yeates et al., 2018, 2021). In Fig. 1, for example, candidates judged weaker overall are advantaged in their scoring if they are examined by Examiner 1 compared to Examiner 2. The opposite is the case for those rated more highly on the global grades. Station-level standards set using examinee centred methods, using scores and grades to generate cut-scores, are also be impacted (Homer, 2020)—this includes assessments that use the well-known borderline regression method (Kramer et al., 2003; McKinley & Norcini, 2014).

One begins to see in Fig. 1 that a traditional conception of examiner stringency based solely on domain scores (or solely on grades) would not capture the essential qualitative differences in examiner behaviour it illustrates. It would also not necessarily allow for the full impact on cut-scores, and hence on assessment outcomes, to be properly investigated. To better understand and measure the impact what is going on here, we need an approach to examiner stringency that uses both scores and grades *simultaneously*. This paper, therefore, uses a large exam dataset to quantify the extent to which the different patterns of scoring suggested in Fig. 1 exist in real OSCE data, and assesses the extent to which this might impact on pass/fail decisions in stations and at the exam level. The explicit research questions addressed are:

1. How much variation in examiner scores is due to traditional stringency effects, and how much is due to differential discrimination by examiners?
2. What is the impact of differential examiner stringency on assessment decisions—at the station and exam level?

The paper continues by outlining the exam context, candidate sample and statistical methods used in the work. The findings then evidence how much variation in scores can be attributed to both conceptualisations of examiner stringency, and to estimate what effect this might have on pass/fail decisions. The work concludes with a discussion of what this all means for OSCE practice in terms of assessment design and scoring, and the managing of examiners.

### Exam context

The data in this study is from the UK Professional and Linguistic Assessments Board OSCE (known as PLAB2), an 18 station summative clinical assessment for international medical graduates who want to begin working in the National Health Service in England (General Medical Council, 2020a, 2020b). PLAB2 is set at a level equivalent to that of a post-graduate trainee entering the second year of training (called FY2 in the UK). The assessment is intended to ensure that candidates can apply their medical knowledge in order to provide good care to patients at the FY2 level.

There is a single examiner in each PLAB2 station, and in most stations there is a simulated patient (SP) played by a paid and trained actor. Each station lasts 8 min with 90 s reading time between stations, and typically there are approximately 36 candidates in each exam (i.e. there are usually two sessions in each exam with the same examiner in the same station). All examiners are trained in advance of examining, and on the day of each exam station-level calibration takes place between pairs of proximate examiners (and SPs) to maximise consistency across stations. Further quality assurance is provided via a range of post hoc analysis following the exam.

In the longer term, the exam is overseen by a highly experienced assessment panel of 30–40 senior clinicians responsible for developing new stations and driving PLAB2 assessment practice. Annual reports are produced to further enhance confidence in the exam and its outcomes (General Medical Council, 2022), and a range of research around PLAB2 has demonstrated reasonable reliability (Homer, 2022) and discussed standard setting and other issues (Homer, 2020, 2023).

In terms of scoring, the outcomes awarded by the single examiner in each PLAB2 station are as follows:

- *A total domain score* on scale from 0 to 12. This is the sum of three separate domain scores (each scored 0 to 4) in
  - *Data gathering*, *technical and assessment skills.*
  - *Clinical management skills*
  - *Interpersonal skills*
  In each domain, there are positive and negative station-specific key feature descriptors to guide examiners in their judgments.
  To aid interpretation, in this paper all domain scores are converted to the percentage of the maximum (12) available.
- *A single global grade* on a scale from 0 to 3 providing an overall holistic judgment of candidate performance in the station as follows:
  - 0 = *fail*: could not carry out work of a day one FY2.
  - 1 = *borderline*: not convinced could carry out work of a day one FY2.
  - 2 = *satisfactory*: could carry out work of a day one FY2 safely.
  - 3 = *good*: could be expected to carry out work of a day one FY2 to a high standard.

As is standard OSCE practice (Khan et al., 2013), these two measures of the performance are awarded independently by the examiner, according to their interpretation of the relevant rubrics in place.

Borderline regression is used for station-level standard setting in PLAB2 (Kramer et al., 2003; McKinley & Norcini, 2014). The aggregate of these plus a standard error of measurement based on Cronbach’s alpha for domain scores (Hays et al., 2008) gives the overall exam-level cut-score for each separate PLAB2 exam.^1^ The overall pass rates for PLAB2 over the period of the study are approximately 72% at the station-level, and 84% at the exam-level.

### Exam data

The data were generated prior to the COVID pandemic (2016–2019), and consist of 313,593 rows of data from 442 separate PLAB2 examinations—one row for each candidate/station interaction, with a median of 612 rows of data for a single examination.

There is a unique code for each level of each facet of the exam—candidate (n = 17,604), station (n = 390), examiner (n = 862) and exam (n = 442). Whilst in a single exam, examiners are nested in stations, each level of examiner and all other facets repeat in the data across exams, typically many times.—For example, the median occurrence across the full dataset for a particular candidate, examiner, station and exam are 18, 204, 707, and 612 respectively. Typically examiners are allocated to different stations in different PLAB2 examinations with no underlying pattern. With an appropriate methodology (next section), this unbalanced design allows for estimation of all main effects for these facets on scoring in the data.

## Methods

### Statistical modelling of total domain scores in stations

The main statistical approach is to use linear mixed models to separate out variance in station-level total domain scores due to the independent effects of global grade, candidate, station, and examiner, treating each of these three latter facets as random effects (Bell et al., 2019). The R package lme4 (Bates et al. 2015; Crowson, 2020) is used to do this. Other data manipulation and analysis is carried out in IBM SPSS (IBM Corp, 2021).

Under such an approach, candidates, stations and examiners are allowed to vary in their ability/difficulty/stringency respectively (in total domain scores) having taken account of the impact of candidate global grades on domain scores.

Importantly, in the modelling examiners are also allowed to vary in their discrimination across grades via the inclusion of a random slope for examiners across grades—this allows precisely for estimation of the differential examiner behaviour witnessed in Fig. 1.

In pseudo-code, the statistical model can be represented as follows with further explanation immediately following^2^^,^^3^:

$$\begin{aligned} {\text{TOTAL}}\_{\text{DOMAIN}}\_{\text{SCORE}} & = {1} + {\text{GLOBAL}}\_{\text{GRADE}} + \left( {{1}|{\text{CANDIDATE}}} \right) \\ & \quad + \left( {{1}|{\text{STATION}}} \right) + \left( {{1} + {\text{GLOBAL}}\_{\text{GRADE}}|{\text{EXAMINER}}} \right) \\ \end{aligned}$$

A standard intercept is estimated (via the ‘1’ in the equation after the equals sign), and the global grade is treated as a fixed effect (i.e. a continuous regression predictor).

The notation (1| FACET) indicates FACET is being treated as a random effect—this provides a single estimate (known as a random intercept) for each level of FACET having taken into all other predictors (fixed and random effects) in the model. For candidates this uses all the data to produce a modelled version of their score (i.e. their ability), and for stations it is a measure of the typical score in the station (i.e. how easy the station is).

The final part of the model, (1 + GLOBAL_GRADE | EXAMINER), does two things:

1. As with candidates and stations, this estimates examiner (traditional) score stringency (i.e. what kind of score they typically give—with lower scores indicating more stringent examiner behaviour and vice versa).
2. It also estimates a random slope for examiner by global grade—in other words, the modelling allows examiners to vary in their slope across grades—with higher values corresponding to more discriminating examiners.^4^

Taken together, these two estimates allow for the modelling of the examiner behaviour hypothesised in Fig. 1.

With the inclusion of global grades in the modelling of station-level domain scores, we might hypothesise that we would not expect candidate and station factors to explain that much variation in these. This is because once you know the candidate grade in a station, the total score has a degree of predictability regardless of which candidate or which station it is—imagine Fig. 1 repeated with the same examiners but at a different station, or with different candidates—presumably the picture would be somewhat similar.

However, there is more uncertainty in what we might expect of the examiner random intercept and random slope effects given earlier work underlying how much variation in scores and grades examiners contribute (Homer, 2020, 2022).

### ‘Fair’ scores, cut-scores and pass/fail outcomes

Having run the mixed model and estimated all effects, we can construct what we might refer to as a model-based fair score for each candidate in each station. This is what the model predicts the candidate would score given their observed (i.e. actual) grade in the particular station if they had been examined by the typical (i.e. ‘fair’) examiner rather than the actual examiner present. We can also then produce observed and modelled station- and exam-level cut-scores using borderline regression on observed and fair scores respectively. We can compare these repeated measures on candidates using the paired-test.

Finally, pass/fail decisions derived using observed scores (and cut-scores from these) can be compared with those derived from fair scores. This allows us to estimate the full effect of differential examiner stringency, as conceptualised in Fig. 1, on PLAB2 outcomes across the full dataset. The McNemar test is used to test whether there are statistically significant differences between the patterns of outcomes for observed pass/fail decisions and those based on fair scores/cut-scores.

## Results

### Statistical modelling of total domain scores in stations

The estimates for the fixed effects and variance components for the random effects are shown in Table 1.^5^

**Table 1:** Fixed and random effects for model with TOTAL_DOMAIN_SCORE as outcome

Predictor	Estimate (SE)	t-value
*Fixed effects*		
Intercept	30.96 (0.26)	120.3
GLOBAL_GRADE	17.51 (0.11)	160.9

Facet	Variance	Variance (%)
*Random effects*		
CANDIDATE (intercept)	3.94	2.9
STATION (intercept)	3.23	2.3
EXAMINER (intercept)	46.25	33.5
EXAMINER (slope for GLOBAL_GRADE)	9.36^a^	6.8
Residual	75.08	54.5
Total	137.86^b^	100.0
Number of observations = 313,593; CANDIDATE = 17,604; STATION = 390; EXAMINER = 862		

^a^The correlation between EXAMINER intercept and slope estimates was − 0.62. This is not surprising, given that an examiner with a higher intercept (e.g. Examiner 1 in Fig. 1) is more likely to have a lower slope and vice versa, given that total scores are bounded (i.e. cannot exceed 100%)

^b^The total variance can be thought of informally as measuring the variation that remains once the fixed effects have been accounted for (i.e. around the line of best fit based on the fixed effects - in this case, Intercept and GLOBAL_GRADE)

For the typical candidate in a typical station with a typical examiner, the fixed effects are interpreted as follows:

- Intercept is the model-based percentage score that the candidate would score at the fail grade i.e. 31.0%
- GLOBAL_GRADE gives the difference in total percentage domain scores across successive global grades (i.e. 17.5% between fail and borderline, or borderline and satisfactory, or satisfactory and good).

Moving on to the random effects, we see that over a third (33.5%) of the variance in total domain scores is accounted for by the EXAMINER (intercept) facet, and an additional 6.8% by the variation in the EXAMINER (slope) facet. In other words, examiners remain important in influencing station-level scores even having taken into account global grades, and they do this in two distinct ways (as was originally hypothesised in Fig. 1). This analysis essentially answers our first research question.

As was also previously hypothesised, little variance in domain scores is accounted for by either CANDIDATE or STATION facets (2.9% and 2.3% respectively, again once global grades are taken into account).

Just over half of variance in scores is not explained by the model (residual = 54.5%). This tells us that there is a lot of variation in station-level total domain scores that is not captured by the factors as included in the model. In other words, the candidate outcome in a station is far from entirely predictable even once you know their underlying ‘overall ability’, their grade, the examiner and the station.

**Fig. 2:**
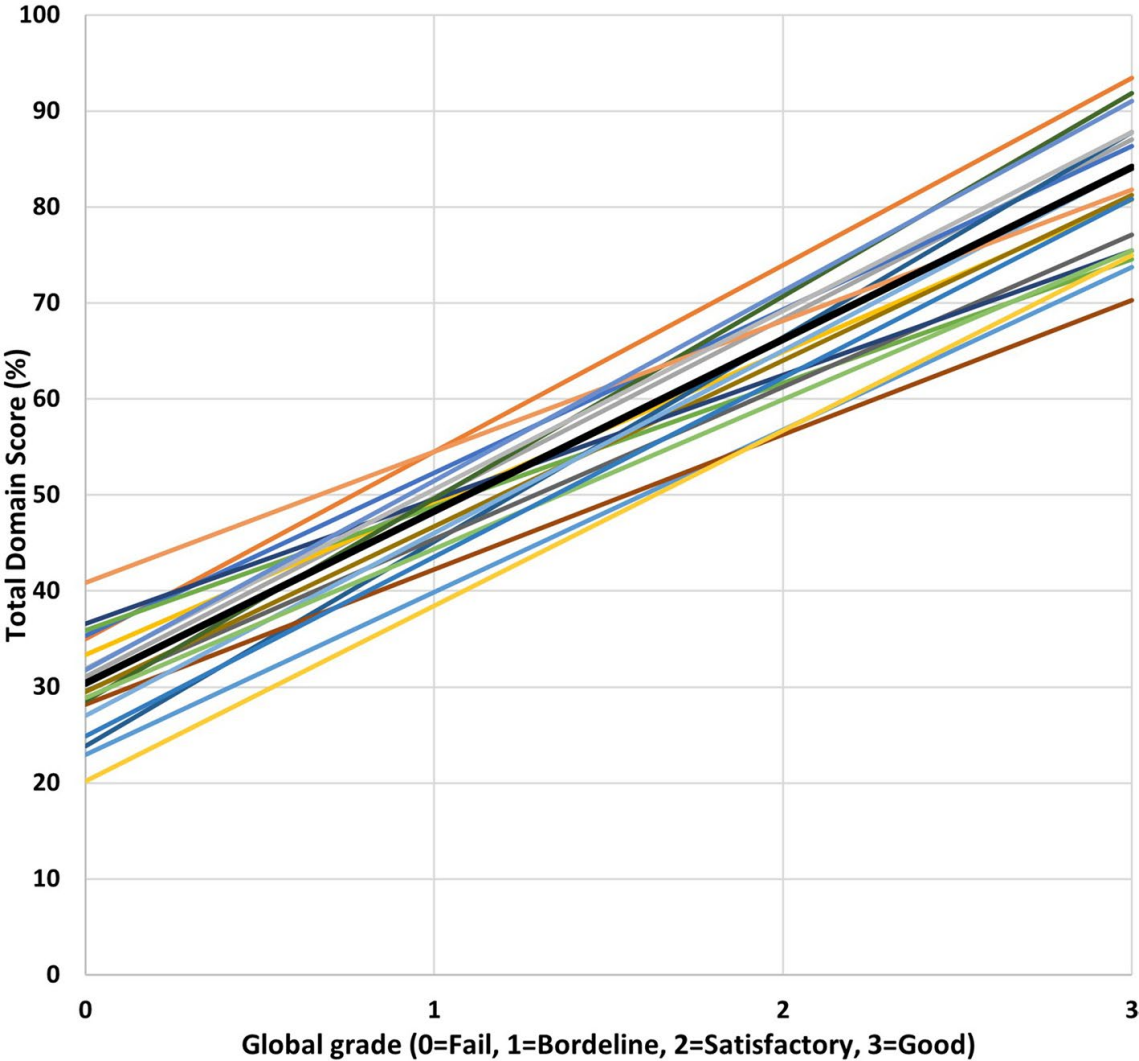
A random selection of 20 examiners (and the typical examiner)

To illustrate variation across examiners, Fig. 2 shows the equivalent of Fig. 1 for a random sample of 20 real PLAB2 examiners based on actual estimates from the model (i.e. 20 specific individual examiner intercepts and slopes). The bolded black line uses the fixed effect intercept and grade and represents the typical examiner in the typical station for the typical candidate.

We observer in Fig. 2 that the vertical spread between lines is generally a greater influence on total domain scores than is the difference in slopes—and this is consistent with the magnitudes of the two variance component estimates in Table 1—EXAMINER (intercept) and EXAMINER (slope for GLOBAL_GRADE).

### ‘Fair’ scores, cut-scores and pass/fail outcomes

Table 2 summarises the comparison between observed candidates scores and fair scores (station level, first data row; exam level, third data row). It also summarises the difference in BRM cut-scores when using observed and fair candidate scores (station level, second data row; exam level, fourth data row).

**Table 2:** Difference between observed and fair percentage scores and cut-scores

Comparison	n	Difference in percentage scores: observed—fair						
		Mean	5th, 95th percentiles	Standard deviation	t	Degrees of freedom	p-value	Cohen’s d
Station-level candidate scores: observed—fair percentage scores	313,593	− 1.0%	− 16.9%, 16.4%	10.4%	− 56.0	313,592	< 0.001	0.10
Station-level BRM cut-scores: observed—fair percentage scores	7877^a^	− 1.5%	− 10.5%, 8.9%	5.9%	–22.5	7876	< 0.001	0.25
Exam-level candidate: observed—fair total percentage scores	17,604	–1.0%	− 4.8%, 2.9%	2.3%	− 58.8	17,603	< 0.001	0.44
Exam-level BRM cut-scores: observed—fair total percentage scores	442	− 1.1	− 3.7%, 1.4%	1.6%	− 15.0	441	< 0.001	0.71

^a^There are 7877 individual OSCE station administrations across the 442 exams in the data

We see that fair scores and fair cut-scores are typically higher than their observed counterparts (all values in the ‘mean’ column, observed—fair, are negative). Table 2 also shows that the difference in cut-scores (observed—fair) is slightly larger than that in candidate scores at both station and exam level. As we might expect, the standard deviation of differences between observed and fair values are smaller in the exam-level analysis compared to that at the station level. The final column of Table 2 gives the measure of the effect size for the paired difference (Cohen’s d). This is an indication of the size of the average difference in the comparison of means—with common guidelines of d = 0.2 as ‘small’, d = 0.5 as ‘medium’ and d = 0.8 as ‘large’ although these classifications are recognised as somewhat arbitrary (Cohen, 1988; Thompson, 2007).

To explicitly begin to answer RQ2, we now compare pass/fail decisions at the station level between those derived using fair observed scores/cut-scores and those derived from the observed data. In Table 3, we see that 89.5% (= 22.0% + 67.4%) of station-level candidate decisions are the same across both, but that the remainder (10.5%) are not—this is a statistically significant change in the overall pattern of pass/fail decisions (McNemar, p < 0.001). There are slightly more passes using the fair scores/cut-scores compared to using the observed scores/cut-scores (station-level pass rates 73.3% and 72.2% respectively).

**Table 3:** Comparison of pass/fail decisions at the station level—observed and fair

	Fair scores/cut-scores		Total
	Fail	Pass	
*Observed scores/cut-scores*			
Fail			
Count	69,019	18,274	87,293
% of Total	22.0%	5.8%	27.8%
Pass			
Count	14,788	211,512	226,300
% of Total	4.7%	67.4%	72.2%
*Total*			
Count	83,807	229,786	313,593
% of Total	26.7%	73.3%	100.0%

The equivalent analysis at the exam level is shown in Table 4,^6^ where overall misclassification of candidates is at a lower rate than at the station level but still represents a significant difference in patterns of percentages between observed and fair (McNemar, p < 0.001).

**Table 4:** Comparison of pass/fail decisions at the exam level—observed and fair

	Fair scores/cut-scores		Total
	Fail	Pass	
*Observed scores/cut-scores*			
Fail			
Count	2178	574	2752
% of Total	12.4%	3.3%	15.6%
Pass			
Count	69	14,783	14,852
% of Total	0.4%	84.0%	84.4%
*Total*			
Count	2247	15,357	17,604
% of Total	12.80%	87.20%	100.00%

We see that there is approximately a 3-percentage point higher exam level pass rate for fair scores compared to observed (87.2% versus 84.4%). Most of this difference comes from the 3.3% who failed based on observed scores but passed on fair scores. Very few candidates (0.4%) pass the exam based on observed scores but would have failed on fair scores.

## Discussion

### A new conceptualisation of examiner stringency

This work adds to the literature around differential examiner stringency (Bartman et al., 2013; Harasym et al., 2008; McManus et al., 2006; Yeates et al., 2020) by developing a more nuanced conceptualisation of it in contexts where examiners use both checklist/domain scoring and global grades to assess performance in OSCEs. In essence, the new evidence suggests that examiner behaviour is systematically more complex than is often discussed in the literature. Oversimplistic conceptualisations of examiner stringency could be problematic when, for example, seeking to correct for differential stringency (‘error’)—this issue needs great care, and might do more harm than good in many contexts.

The work uses a rich exam dataset to demonstrate that a traditional view of examiner stringency, where variation across examiners is measured solely in terms of scores in stations, can fail to capture a key additional element of stringency—variation in discrimination across global grades (as illustrated empirically in Fig. 2). This additional component of stringency is smaller in magnitude than the traditional component (7% and 34% of total station score variance, respectively), but it is responsible for far more variance in scores at the station level than are stations and candidates (2% and 3% respectively—once global grades are taken into account) (Table 1). This might seem counterintuitive at first, especially given what we know about case/context specificity where performance on one task does not always predict well performance on another (Norman et al., 2006; Schauber et al., 2018). However, the inclusion in the modelling of global grade as a predictor ensures that stations or candidates do not impact *additionally* very much on systematic variation in total scores at the station level.

That examiners still do have an important impact on scoring even when global grades are accounted for is a key finding of the current work, whilst the finding that just over a half of the variance is not explained by the factors in the model does suggest that there might well be other unmeasured factors, or just idiosyncratic examiner behaviours or bias, that the modelling as implemented does not capture (Wong et al., 2023). It is possible, even likely, that the relatively large residual is also to an extent a result of the model estimating a single, fixed value for candidate ability, when case/context specificity might suggest that candidates will vary in performance across stations. However, the relatively limited data on individual candidates in the exam data (typically 18 rows of data, 1 per station) makes it unlikely that a more complex analysis that allowed for candidate estimates to vary across cases would be possible.

### Impact on pass/fail decisions

Measurement error in scores is likely to produce error in pass/fail decisions (Livingston & Lewis, 1995), and this study gives an indicative quantification of how much difference this new conceptualisation of examiner stringency makes in terms of passing and failing candidates—at both the station and exam level (Tables 3 and 4 respectively). As we might expect, error due to differential examiner stringency is weaker at the exam level than at station level, but there remain around 4% of candidates mis-classified overall (i.e. false positives or false negatives at the exam level—candidates passing or failing in error, respectively). This is a smaller effect than that found in other work (Homer, 2022; Yeates et al., 2018, 2021). However the methods employed are quite different—for example, in the Yeates and colleagues’ work (2018, 2021), videoed stations are used to standardise and allow for equating of effects across different student groups. The Homer (2022) work brings with it a different key assumption—in the current study the global grade is assumed in the modelling to be error free (as a regression predictor) whereas in the earlier work, this was not the case since grades were treated then as an outcome variable, thereby allowed to be measured with error (Nimon, 2012).

Homer (2022) found a systematic bias in borderline regression standards with ‘fair’ cut-scores lower typically by 3% or so compared to those derived from observed data. In the current work this bias is in the opposite direction and smaller (≈1%, Table 2). These differences in findings underline the important message that modelling assumptions really matter, and that different methodological approaches, even using the same or similar data, can lead to different, even contradictory, findings. That said, in the previous modelling work (Homer, 2022), scores and grades were treated separately in the analysis, whereas in the current work the two measures of performance are used simultaneously throughout the analysis—and this is likely a relative strength of the current approach. The issue of correcting for ‘error’ and the impact this might have on assessment outcomes requires further research, possibly using simulation approaches to model complex examiner behaviour in particular (Morris et al., 2019).

Returning to the issue of the impact on pass/fail outcomes, there is a debate to be had about the extent to which correcting for differential examiner stringency to produce ‘fair’ scores can or should be acted on in individual cases. As already discussed, all modelling brings its own assumptions (Nimon, 2012), and all modelling estimates are derived across groups of cases rather than being assumed appropriate for use at the individual candidate level, especially in high stakes settings. Hence, it is arguably better to regard all outcomes of modelling as not specifically applicable to individuals, whereas across the full sample the analysis remains robust in terms of estimating the general effect of different facets on scoring. In this study, this is particularly true for examiners, given that the amount of data for them far exceeds that on each candidate—the median occurrence in the data of each examiner is over 200, compared to only 18 for each candidate. This implies that examiner effects are much more likely to be better estimated than those for candidates, and we would argue that this type of psychometric analysis does indeed provide insightful knowledge about examiner behaviour in particular (Pearce, 2020; Schauber et al., 2018). There is somewhat of an irony here, given that the main purpose of assessment is usually to sort or classify candidates (McKinley & Norcini, 2014) rather than to provide measures of other facets of the examination set-up such as examiner.

One limitation of the study is that it models a single main effect for all examiners, and does not, for example, allow examiner stringency to vary across stations or station types—there is simply insufficient data on all examiners to do this. However, given the relatively large amount of data available on some individual examiners, it might be possible to develop in the future a more complex examiner effect on a subset of frequently occurring examiners to allow for such interaction effects. A second limitation in this work is that it did not consider the issue of systematic bias in examiner scoring, for example, bias by candidate gender—that would require additional data and methods (for example differential item (i.e. station) functioning) (Osterlind & Everson, 2009). The distinction between issues of examiner stringency and examiner ‘bias’ are worthy of wider consideration in the literature.

### Informing practice

How best can this work inform OSCE practice? One clear and consistent implication of this and other related work (Homer, 2020, 2022; Yeates et al., 2018, 2021), is the need to ensure that examiner effects are ameliorated through sufficient sampling across examiners—usually most effectively by ensuring that candidates experience enough stations to be sufficiently confident in the exam outcomes (Khan et al., 2013) via adequate sampling of the key source of ‘error’, the examiner.

In terms of OSCE examiner training and development, it might be helpful to keep emphasising to examiners that scoring, grading and the relationship between them really matter, especially under examinee-centred approaches to standard setting such as borderline regression. The key message to examiners might be that it is more complicated than just considering in isolation your scoring as higher (or lower) than examiner peers—it also matters how you produce global grades in relation to station scores. Illustrating the impact of different or archetypal patterns of scoring/grading, such as those shown in Fig. 1, might help to reduce unwanted variation by examiners. However, the existing evidence suggests there are many complex issues that impact on how examiners judge performance regardless of the type of instrument used to capture those judgments (Wong et al., 2023; Yeates et al., 2013). It might be that we do have to accept that individual examiner judgments are always partly subjective (Hodges, 2013; Valentine et al., 2022) whilst designing our summative assessments robustly enough to deal with this through sufficient sampling across examiners and stations.

## Data Availability

For reasons of confidentiality and assessment security, the data used in this study is not openly available to researchers or the public.

## Footnotes

1 PLAB2 also has a minimum station hurdle requirement (Homer, 2023) but this has been omitted to keep the analysis and arguments more focussed.

2 EXAM is not included as a separate facet – the (additional) effect of EXAM is small (Homer 2022) leading to estimation problems in the current study.

3 The actual R code snippet for the model using lme4 (Bates et al. 2015; Crowson 2020) is as follows: model <—lmer(STATION_DOMAIN_SCORE_PERCENTAGE ~ 1 + EXAMINER_OVERALL_JUDGEMENT + (1|CANDIDATE) + (1|STATION) +  + (1 + EXAMINER_OVERALL_JUDGEMENT | EXAMINER), data = PLAB2_Data).

4 This also allows the two examiner random effects (intercept and slope) to be correlated rather than forcing them to be uncorrelated.

5 The model residuals were approximately normally distributed with (5th, 95th) percentile = (-13.7%, 14.1%) on the total domain score percentage scale, and a standard deviation of 8.5%. This indicates acceptable model fit.

6 Note, the exam level cut-scores include the addition of 1 standard error of measurement, based on Cronbach’s alpha – which is typically of the order of 3.8% and 3.5% using observed and fair scores respectively.
